# KAP survey on integrated traditional Chinese and western medicine for ischemic stroke among healthcare workers in Zhengzhou City: A cross-sectional study based on multi-tiered healthcare institutions

**DOI:** 10.1097/MD.0000000000046069

**Published:** 2025-11-28

**Authors:** Yiwei Zhang, Haobo Gao, Hongtu Tan, Jiabin Wang, Guofang Yang, Dongyi Yang, Yaheng Zhao, Lei Xie, Tao Wu

**Affiliations:** a Interventional Department, Brain Disease Diagnosis and Treatment Center, The First Affiliated Hospital of Henan University of Chinese Medicine, Zhengzhou, Henan Province, China; b The First Clinical College of Henan University of Chinese Medicine, Zhengzhou, Henan Province, China; c Henan Collaborative Innovation Center for the Prevention and Treatment of Major Diseases with Traditional Chinese and Western Medicine, Zhengzhou, Henan Province, China.

**Keywords:** acute ischemic stroke, cross-sectional questionnaire survey, integrated traditional Chinese and Western medicine treatment, knowledge-attitude-practice model, science and technology program for public benefit

## Abstract

This cross-sectional study (n = 517) assessed healthcare workers’ knowledge, attitudes, and practices (KAP) regarding integrated traditional Chinese and Western medicine (TCM-WM) for acute ischemic stroke (AIS) across 12 multi-tiered institutions in Zhengzhou. Using validated questionnaires and multivariate logistic regression, we identified tertiary hospital staff, integrated TCM-WM practitioners, and senior professionals as KAP strongholds (odds ratio (OR) ≥ 2.7, *P* < .001). Community hospitals and nursing staff exhibited knowledge deficits but strong operational competence. Knowledge-practice correlation was robust (*R* = 0.612), mediated by professional title and experience. Findings underscore the need for tiered training systems, role-specific competency frameworks, and evidence-based protocols to standardize TCM-WM integration in regional stroke networks. In the treatment of AIS, challenges such as the limitation of the thrombolytic time window and insufficient neurological functional recovery persist. The integrated traditional Chinese and Western medicine model demonstrates potential through multi-target interventions, yet there are disparities in healthcare professionals’ understanding and practice of this approach, along with a lack of regional empirical evidence. This study aims to evaluate the KAP of healthcare professionals in Zhengzhou regarding the integrated traditional Chinese and Western medicine treatment for AIS, identify influencing factors, and provide evidence-based support for optimizing the regional stroke prevention and treatment network. A cross-sectional survey was conducted from January to March 2024. Using a multi-stage, stratified sampling method, 586 healthcare professionals from 12 medical institutions in Zhengzhou (including tertiary, secondary, and community hospitals) were enrolled in this study. The questionnaire covered basic information, AIS knowledge assessment (33 items), treatment attitudes (12 items), and practice behaviors (10 items). SPSS 26.0 was used for descriptive statistics, correlation analysis (Pearson), and multiple logistic regression analysis. Of the 586 questionnaires distributed, 517 were validly returned, yielding an overall valid response rate of 88.23% (517/586). Stratified analysis by demographic characteristics was performed on the KAP scores of the 517 medical staff. Tertiary hospitals exhibited significantly better KAP scores than community hospitals (Knowledge OR = 2.74, Practice OR = 2.38, *P* < .05). Integrated TCM-WM practitioners demonstrated the highest KAP scores (Knowledge OR = 3.25, *P* < .05), while nursing staff had the lowest knowledge scores (23.13 ± 3.67) but outstanding practical competency (40.13 ± 5.57). Senior professional titles were associated with higher knowledge (OR = 3.95), and staff with >15 years of experience showed 2.89 times higher practice initiative than those with <5 years (*P* < .05). Among physicians, TCM majors showed significant KAP advantages (Practice OR = 5.22 vs Clinical medicine, *P* < .05), and male physicians exhibited higher practice initiative (OR = 1.88, *P* = .017). Among other medical staff (nurses/technicians), females demonstrated stronger practice initiative (OR = 2.05, *P* = .008), and intermediate titles were associated with more positive attitudes (OR = 2.10, *P* = .028). The study revealed that healthcare professionals in Zhengzhou generally possess a sound understanding of integrated traditional Chinese and Western medicine for AIS treatment. However, disparities were observed across healthcare institution tiers and professional specialties. It is recommended to establish a tiered training system, enhancing knowledge dissemination among community hospitals and junior staff. Treatment protocols should leverage the specialized strengths of integrated medicine to design comprehensive plans, while the practical competencies of nursing staff can be utilized to develop knowledge translation tools. Future research should validate the effectiveness of these tiered strategies through intervention trials and explore the neurocognitive mechanisms underlying a direct “knowledge-to-practice” pathway.

## 1. Introduction

Acute ischemic stroke (AIS), the second leading cause of death globally and the primary cause of adult disability, imposes a significant disease burden in China.^[1]^ Epidemiological data indicate^[2]^ approximately 2.4 million new stroke cases occur annually in China, with AIS accounting for over 70%. The incidence continues to rise at an annual rate of 8.7%, showing a trend towards affecting younger populations. Although the widespread adoption of intravenous thrombolysis and endovascular mechanical thrombectomy has significantly improved vascular recanalization rates, strict therapeutic time windows (thrombolysis ≤ 4.5 hours, thrombectomy ≤ 6 hours) limit timely reperfusion treatment to <20% of patients.^[3,4]^ Furthermore, numerous studies have found^[5,6]^ that even with successful recanalization, approximately 50% of patients still suffer severe functional disabilities, highlighting the limitations of Western medicine alone in neuroprotection and functional rehabilitation. In recent years, integrated TCM-WM treatment models have offered new pathways to overcome AIS treatment bottlenecks. Basic and clinical research has progressively revealed the multi-target intervention mechanisms of TCM, such as herbal medicines promoting blood circulation and resolving blood stasis enhancing collateral circulation, heat-clearing and detoxifying preparations mitigating reperfusion injury, and acupuncture promoting neural functional remodeling. These demonstrate unique advantages synergizing with thrombolysis/thrombectomy in the acute phase and during recovery/rehabilitation.^[7,8]^ Study demonstrates that integrated traditional Chinese and Western medicine therapy can increase reperfusion efficiency by 22% and, in addition, has shown significant effects in improving neurological function scores and reducing complications.^[9]^ The 2023 edition of the Chinese Guidelines for the Diagnosis and Treatment of Acute Ischemic Stroke^[10]^ for the first time included Butylphthalide Sodium Chloride Injection (protecting neurons through anti-inflammatory, antioxidant, and anti-apoptotic effects) as a Class II recommended drug, signifying the endorsement of integrated TCM-WM treatment by evidence-based medicine. By integrating traditional Chinese medicine interventions (such as Chinese herbal medicines for activating blood circulation, resolving stasis, clearing heat, and detoxifying, as well as acupuncture) with Western reperfusion therapies (thrombolysis and thrombectomy), this guideline establishes an integrated “disease-syndrome combination” approach. This model breaks through the limitations of Western-only treatment regarding the restricted time window and neuroprotective efficacy. However, current integrated TCM-WM practice for AIS in China faces challenges including: primary care medical staff’s vague understanding of TCM mechanisms; technological gaps between tertiary and county hospitals; lack of operational consensus on key issues like optimal timing for acupuncture intervention and potential TCM-WM drug interactions. These factors collectively contribute to fragmented and arbitrary implementation of integrated treatment plans at the clinical level, hindering the standardization and reproducibility of therapeutic outcomes.^[11]^ Driven by the “Healthy China 2030” strategy, the 2024 Zhengzhou Science and Technology Benefiting the People Program has funded this survey study. It aims to scientifically assess healthcare professionals’ current knowledge, attitudes, and practices (KAP) regarding integrated TCM-Western medicine therapy for AIS, thereby providing empirical evidence for developing regional integrated pathways and promoting the allocation of high-quality medical resources to primary care and the enhancement of grassroots service capacity.

## 2. Patients and method

### 2.1. Study design and participants

This study selected Zhengzhou City in Henan Province as the research site. As a National Central City, Zhengzhou hosts a resident population of over 12 million and features a typical “pyramid”-shaped distribution of medical resources, supported by a comprehensive three-tier healthcare network. Designated as a key city in the national stroke prevention and treatment system, Zhengzhou has established a city-wide three-tier prevention and treatment network comprising “Stroke Centers – Stroke Prevention and Control Centers – Community Screening Sites.” Consequently, conducting this study in Zhengzhou enables effective capture of the real-world conditions among healthcare professionals across different levels of medical institutions, from top-tier tertiary hospitals to grassroots community health centers. The findings are expected to provide valuable insights for developing regional stroke prevention and treatment systems in similar cities across China.

A cross-sectional survey design was adopted and implemented from January to March 2024 across 12 medical institutions in Zhengzhou (comprising 4 tertiary hospitals, 4 secondary hospitals, and 4 community hospitals). Inclusion criteria were: practicing physicians or nurses clinically engaged in neurology, emergency medicine, traditional Chinese medicine, or rehabilitation departments within the target institutions; direct involvement in the diagnosis, treatment, nursing care, or rehabilitation of patients with AIS; and provision of informed consent and voluntary participation. Exclusion criteria included: healthcare workers in non-clinical roles (e.g. administrative, logistical, or technical support); those on leave or extended external training during the study period; and interns and visiting trainees.

A multi-stage, stratified random sampling method will be employed to recruit 586 frontline clinical physicians or nurses in Zhengzhou for the questionnaire survey. Stratification will be based on hospital level (Tertiary: Secondary: Community = 4: 4: 4).

### 2.2. Survey instrument

The structured questionnaire was developed based on the WHO KAP model, referencing international management guidelines for AIS^[12]^ and similar research tools.^[13]^ The draft questionnaire was independently reviewed by 2 senior neurologists, and ambiguous or questionable items (e.g. duplicate questions on TCM interventions) were revised to ensure content validity. The final version comprised 4 sections:

1. Demographic characteristics (7 items): Age, Gender, Hospital Level (Tertiary/Secondary/Community), Professional Category (Clinical Medicine/Traditional Chinese Medicine/Integrated TCM-WM/Nursing), Professional title (Senior = Attending Physician/Chief Nurse or above; Intermediate = Resident physician/Charge nurse; Junior = Physician Assistant/Nurse; Assistant Level = Intern/Trainee), Years of practice.
2. Knowledge dimension (33 items): covered pathogenesis (e.g. “atherosclerosis is the main etiology”), therapeutic windows (thrombolysis ≤ 4.5 hours, thrombectomy ≤ 6 hours), TCM application (Indications for herbal medicine/acupuncture), and complication management (e.g. hemorrhagic transformation recognition). Scoring: correct answer = 1 point, incorrect/unclear = 0 points. Total score range: 0 to 33. A higher score is indicative of a better grasp of knowledge on integrated AIS therapy.
3. Attitude dimension (12 items): utilized a Likert five-point scale (1 = strongly disagree, 5 = strongly agree), focusing on 3 aspects: perceived treatment necessity (e.g., “thrombolysis/thrombectomy improves quality of life”); Value of TCM-WM integration (e.g., “combined therapy is superior to monotherapy”); Training willingness (e.g., “willing to learn TCM knowledge”). Total score range: 12 to 60. A higher score is indicative of a more positive attitude toward integrated AIS therapy.
4. Practice dimension (10 items): utilized a frequency-based five-point scale (1 = never, 5 = always), assessing 2 types of behaviors: Clinical practice (e.g. “actively recommend TCM”); Patient education (e.g. “explain the benefits of acupuncture”). Total score range: 10 to 50. A higher score is indicative of more frequent and proactive clinical practice of integrated AIS therapy.

### 2.3. Validity and reliability of the survey instrument

Prior to the formal survey, we assessed the reliability and validity of the questionnaire.

#### 2.3.1. Content validity

Five experts (three chief physicians of neurology and 2 professors in the field of integrated traditional Chinese and Western medicine) were invited to evaluate the questionnaire items. The calculated scale-level content validity index was 0.92, and the item-level content validity indices (item-level content validity index) ranged from 0.80 to 1.00, indicating good content validity of the questionnaire.

#### 2.3.2. Construct validity

Exploratory factor analysis was conducted to validate the structure of the attitude and practice scales. The Kaiser–Meyer–Olkin measure was 0.871, and Bartlett’s test of sphericity was significant (*P* < .001). Using the varimax rotation method, common factors with eigenvalues >1 were extracted, which cumulatively accounted for 68.5% of the total variance, demonstrating good construct validity.

#### 2.3.3. Reliability

Internal consistency reliability (using Cronbach alpha coefficient) and test-retest reliability were evaluated. The overall questionnaire’s Cronbach alpha coefficient was 0.89. Two weeks after the initial survey, 50 participants were randomly selected for a re-test, and the intraclass correlation coefficient was calculated. The intraclass correlation coefficient values for the knowledge, attitude, and practice dimensions were 0.91, 0.87, and 0.89, respectively, indicating excellent reliability of the questionnaire.

### 2.4. Data collection and quality management

#### 2.4.1. Questionnaire distribution process

The researchers first contacted the heads of the Medical Affairs Office or Nursing Department at the 12 target hospitals to explain the study’s purpose and procedures. Upon obtaining approval, these designated liaisons disseminated the recruitment notice, which contained the questionnaire link and QR code, to the work groups of eligible healthcare professionals via the head nurses or department directors of relevant clinical departments (e.g., Neurology, Emergency Medicine).

#### 2.4.2. Participant task

Participants scanned the QR code or clicked the link to access the survey. After reading the electronic informed consent form and providing their confirmation, they could proceed to complete the questionnaire.

#### 2.4.3. Completion timeline and reminders

The data collection window was open from January 15 to March 15, 2024. Reminder notifications were sent through the hospital liaisons once at the 2-week and 4-week marks following the survey launch to encourage participation.

##### 2.4.3.1. Technical quality control

The platform was configured to allow only one submission per IP address to prevent duplicate entries. Mandatory checks for all required items were enforced before submission to ensure completeness. For partially completed questionnaires, the platform automatically saved progress, allowing participants to resume within a set period (7 days) from the same device (relying on browser cache). Participants could return within the same browser session to modify previous answers, but changes were prohibited after final submission. Skipping mandatory questions was not permitted. Based on pre-testing results, the estimated average completion time for the entire questionnaire was 15 to 20 minutes.

##### 2.4.3.2. Invalid questionnaire identification and exclusion

Questionnaires exhibiting clear patterned responses across all items were flagged by a backend algorithm. Examples of invalid patterns included selecting the same option for all multiple-choice questions (e.g., consistently choosing “1” [Strongly Disagree] on all Likert 5-point scales, or marking “True” for all statements in the knowledge section). A total of 15 such questionnaires were identified and, after manual verification, excluded from the final analysis.

### 2.5. Sample size

Based on the methodology commonly employed in quantitative surveys, the sample size was determined to be 5 to 10 times the number of KAP items. Given that this study comprised 62 KAP items, the minimum required sample size ranged from 310 to 620 participants (this represents a semi-quantitative sample size estimation method frequently used in KAP studies). Consequently, a total of 586 questionnaires were distributed in this research.

### 2.6. Statistical analysis

Statistical analyses were performed using SPSS software (Version 26.0; IBM, Armonk). Continuous data are presented as the mean ± standard deviation and were compared using independent *t*-tests or one-way analysis of variance (ANOVA). Categorical data are expressed as frequencies [*n* (%)]. The Pearson correlation model was applied to analyze correlations. For the multiple logistic regression analysis, the dependent variables were defined as binary categorical variables: the High-Knowledge group (≥80% of the total knowledge score, i.e., ≥26.4 points), the Positive-Attitude group (≥mean score of level 4 on the Likert scale, i.e., ≥48 points), and the Active-Practice group (≥75th percentile of the behavior frequency scale score, i.e., ≥37.5 points). The independent variables included all collected demographic characteristics: gender, age, hospital level, professional category, professional title, and years of practice. Multivariable logistic regression was used to identify variables independently associated with KAP outcomes. A likelihood ratio forward selection procedure (with an entry criterion of *P* < .05) was employed to select factors for inclusion in the multivariable logistic regression models. A *P*-value of <.05 was considered statistically significant.

## 3. Results

### 3.1. Survey response rate

A total of 600 questionnaires were distributed to healthcare professionals, with 586 completed questionnaires returned, yielding an overall valid response rate of 88.23%. To assess potential non-response bias, we compared the distribution of key demographic characteristics (hospital level, professional category) between respondents and non-respondents. The χ^2^ test results indicated no significant differences in the distribution of hospital level or professional category between the 2 groups (*P* > .05), suggesting that the final analyzed sample is reasonably representative of the target population. Details are shown in Table 1.

**Table 1:** Survey response rate.

Project	Responded (n = 517)	Not respond (n = 69)	χ^2^-value	*P*-value
Hospital level				
Tertiary	155 (29.98)	19 (27.54)	1.273	.529
Secondary	173 (33.46)	20 (28.99)		
Community	189 (36.56)	30 (43.48)		
Professional category				
Clinical medicine	237 (45.84)	34 (49.28)	1.013	.908
Traditional Chinese Med	62 (11.99)	10 (14.49)		
Integrated TCM-WM	83 (16.05)	10 (14.49)		
Nursing	135 (26.11)	15 (21.74)		

TCM-WM = traditional Chinese and Western medicine.

### 3.2. Demographic characteristics of participants

The baseline characteristics of the 517 eligible participants included in the final analysis are presented in Table 2. The cohort was predominantly composed of females aged 25 to 35, holding junior professional titles, with backgrounds in clinical medicine, and possessing 5 to 10 years of practical experience. The sample demonstrated broad coverage across hospital tiers, with the largest proportion from tertiary hospitals (36.56%), while community hospitals also constituted a significant share (nearly 30%). In terms of professional distribution, clinical medicine and nursing collectively accounted for over 70% (71.95%) of participants. However, the representation of integrated traditional Chinese and Western medicine (16.05%) indicates potential for further growth. For detailed data, please refer to Table 2.

**Table 2:** Demographic characteristics of participants.

Demographic characteristic	*n*	Proportion (%)
Age (yr)		
<25	52	10.10
25–35	207	40.00
36–45	155	30.00
>45	103	19.90
Gender		
Male	181	35.00
Female	336	65.00
Professional title		
Assistant	10	1.93
Junior	243	47.00
Intermediate	179	34.62
Senior	85	16.44
Professional category		
Clinical medicine	237	45.84
Traditional Chinese Med	62	11.99
Integrated TCM-WM	83	16.05
Nursing	135	26.11
Hospital level		
Community	155	29.98
Secondary	173	33.46
Tertiary	189	36.56
Years of practice (yr)		
<5	129	25.00
5–10	181	35.00
11–15	129	25.00
>15	78	15.00

TCM-WM = traditional Chinese and Western medicine.

### 3.3. Overall scores of knowledge, attitudes, and practices

The mean scores for knowledge, attitudes, and practices among the 517 healthcare professionals in this study were (24.51 ± 3.98), (48.05 ± 5.18), and (37.83 ± 5.96) points, respectively.

### 3.4. Knowledge scores and influencing factors

Statistically significant differences in knowledge scores were observed across groups based on age, hospital level, professional category, professional title, and years of practice (all *P* < .05), while no significant difference was found by gender (*P* > .05). Specifically, knowledge scores were significantly higher among personnel in tertiary hospitals compared to those in community hospitals. Professionals in Integrated Traditional Chinese and Western Medicine achieved the highest scores (28.64 ± 2.15), which were significantly superior to all other professional categories. Individuals with senior professional titles scored significantly higher than those with junior titles, and those with more than 15 years of experience significantly outperformed those with <5 years of experience.

Multivariable analysis revealed that working in a tertiary hospital was associated with 2.74 times greater odds (odds ratio (OR) = 2.74, *P* < .001) of being in the high-knowledge group compared to working in a community hospital, while working in a secondary hospital was associated with 1.88 times greater odds (OR = 1.88, *P* = .004). Professionals in Integrated Traditional Chinese and Western Medicine had 3.25 times the odds (OR = 3.25, *P* < .001) of high knowledge scores compared to nursing staff, and those in Traditional Chinese Medicine had 2.03 times the odds (OR = 2.03, *P* = .004). Holding a senior professional title was associated with 3.95 times greater odds (OR = 3.95, *P* < .001) of high knowledge compared to holding an assistant-level title, and having over 15 years of experience was associated with 3.02 times greater odds (OR = 3.02, *P* < .001) compared to having <5 years of experience. For detailed data, please refer to Table 3.

**Table 3:** Knowledge scores and influencing factors.

Demographic characteristic	Knowledge scores	*P*-value	Variable	High knowledge OR (95% CI)	*P*-value
Age (yr)			Age (Ref: <25 yr)		
<25 yr	22.12 ± 4.21	<.001			
25–35 yr	24.18 ± 3.76		25–35 yr	1.42 (0.95–2.13)	.087
36–45 yr	25.03 ± 3.85		36–45 yr	2.15 (1.38–3.35)	.001
>45 yr	25.89 ± 3.52		>45 yr	3.02 (1.83–4.98)	<.001
Gender					
Male	24.71 ± 4.05	.415	Gender (male vs female)	0.87 (0.62–1.22)	.412
Female	24.41 ± 3.95				
Professional title			Title (Ref: Assistant)		
Assistant	21.50 ± 4.01	<.001			
Junior	23.85 ± 4.12		Junior	3.95 (2.08–7.52)	<.001
Intermediate	25.14 ± 3.55		Intermediate	2.87 (1.55–5.31)	.001
Senior	26.89 ± 2.98		Senior	2.11 (1.15–3.87)	.016
Professional category			Prof Cat. (Ref: Nursing)		
Clinical medicine	25.23 ± 3.31	<.001	Clinical medicine	1.27 (0.85–1.89)	.245
Traditional Chinese Med	26.45 ± 2.77		Traditional Chinese Med	2.03 (1.25–3.31)	.004
Integrated TCM-WM	28.64 ± 2.15		Integrated TCM-WM	3.25 (2.02–5.24)	<0.001
Nursing	23.13 ± 3.67				
Hospital level			Hospital level (Ref: Community)		
Community	22.83 ± 4.15	<.001			
Secondary	24.65 ± 3.88		Secondary	1.88 (1.22–2.89)	.004
Tertiary	25.92 ± 3.41		Tertiary	2.74 (1.82–4.13)	<.001
Years of practice (yr)			Years Pract. (Ref: <5 yr)		
<5 yr	23.05 ± 4.18	<.001			
5–10 yr	24.38 ± 3.79		5–10 yr	1.52 (0.98–2.35)	.062
11–15 yr	25.22 ± 3.64		11–15 yr	2.18 (1.37–3.47)	.001
>15 yr	26.06 ± 3.45		>15 yr	3.02 (1.80–5.07)	<.001

CI = confidence interval, OR = odds ratio, TCM-WM = traditional Chinese and Western medicine.

### 3.5. Attitude scores and influencing factors

With the exception of gender (*P* > .05), statistically significant differences in attitude scores were observed across groups based on age, hospital level, professional category, professional title, and years of practice (all *P* < .05). Healthcare professionals in tertiary hospitals demonstrated significantly higher attitude scores than those in community hospitals. Personnel specializing in Integrated Traditional Chinese and Western Medicine exhibited the most positive attitudes (52.69 ± 3.18). Furthermore, individuals with greater seniority (>45 years old) and those holding higher professional titles also registered significantly higher attitude scores.

Multivariable analysis indicated that working in a tertiary hospital was associated with significantly greater odds of having a positive attitude compared to working in a community hospital (OR = 1.96, *P* = .001). Professionals in Integrated Traditional Chinese and Western Medicine had 2.91 times the odds (OR = 2.91, *P* < .001) of a positive attitude compared to nursing staff. Additionally, medical staff over 45 years of age had 2.24 times the odds (OR = 2.24, *P* = .002) of a positive attitude compared to those under 25 years of age. For detailed results, please refer to Table 4.

**Table 4:** Attitude scores and influencing factors.

Demographic characteristic	Attitude scores	*P*-value	Variable	Positive attitude OR (95% CI)	*P*-value
Age (yr)			Age (Ref: <25 yr)		
<25 yr	45.88 ± 5.52	<.001			
25–35 yr	47.65 ± 5.11		25–35 yr	1.28 (0.85–1.92)	.236
36–45 yr	48.62 ± 4.89		36–45 yr	1.76 (1.13–2.74)	.012
>45 yr	49.47 ± 4.73		>45 yr	2.24 (1.36–3.70)	.002
Gender			Gender (male vs female)	0.92 (0.66–1.29)	.632
Male	48.31 ± 5.24	.403			
Female	47.91 ± 5.15				
Professional title			Title (Ref: Assistant)		
Assistant	44.20 ± 5.65	<.001			
Junior	47.12 ± 5.34		Junior	3.42 (1.81–6.47)	<.001
Intermediate	48.95 ± 4.78		Intermediate	2.54 (1.38–4.68)	.003
Senior	50.88 ± 4.12		Senior	1.98 (1.08–3.63)	.027
Professional category			Prof Cat. (Ref: Nursing)		
Clinical Medicine	48.52 ± 4.59	<.001	Clinical Medicine	1.31 (0.88–1.95)	.184
Traditional Chinese Med	50.21 ± 3.79		Traditional Chinese Med	1.97 (1.22–3.19)	.006
Integrated TCM-WM	52.69 ± 3.18		Integrated TCM-WM	2.91 (1.82–4.65)	<.001
Nursing	46.37 ± 5.11				
Hospital level			Hospital level (Ref: Community)		
Community	46.54 ± 5.52	<.001			
Secondary	48.12 ± 5.03		Secondary	1.42 (0.93–2.17)	.104
Tertiary	49.33 ± 4.61		Tertiary	1.96 (1.30–2.95)	.001
Years of practice (yr)			Years Pract. (Ref: <5 yr)		
<5 yr	46.75 ± 5.48	<.001			
5–10 yr	47.83 ± 5.02		5–10 yr	1.34 (0.87–2.07)	.184
11–15 yr	48.81 ± 4.85		11–15 yr	1.85 (1.16–2.95)	.010
>15 yr	49.72 ± 4.61		>15 yr	2.47 (1.47–4.15)	.001

CI = confidence interval, OR = odds ratio, TCM-WM = traditional Chinese and Western medicine.

### 3.6. Practice scores and influencing factors

Statistically significant differences in practice scores were observed across all demographic characteristics except for gender (*P* > .05), with all other factors showing significant associations (all *P* < .05). Personnel in tertiary hospitals and those specializing in Integrated Traditional Chinese and Western Medicine demonstrated significantly higher practice scores compared to other groups. Notably, although nursing staff had relatively lower knowledge scores, their practice score (40.13 ± 5.57) was at a comparatively high level. Higher professional titles and greater years of practice were also significant positive predictors of proactive practice behaviors.

Multivariable analysis revealed that professionals in Integrated Traditional Chinese and Western Medicine had 3.17 times the odds (OR = 3.17, *P* < .001) of proactive practice compared to nursing staff. The odds of proactive practice were 2.38 times higher (OR = 2.38, *P* < .001) in tertiary hospitals and 1.75 times higher (OR = 1.75, *P* = .009) in secondary hospitals, both compared to community hospitals. Holding a senior professional title was associated with 3.88 times the odds (OR = 3.88, *P* < .001) of proactive practice compared to an assistant-level title. Furthermore, each ascending level in the professional title hierarchy was associated with an approximately 30% increase in the OR.

Clear gradient effects were observed for hospital level (tertiary > secondary > community), professional title (primary > intermediate > senior > assistant), and age (>45 years > 36–45 years). The disparity between knowledge and practice dimensions was more pronounced than that observed in the attitude dimension. Professionals in Integrated Traditional Chinese and Western Medicine significantly outperformed all other specialties across all KAP dimensions (OR > 2.9), while nursing staff emerged as a disadvantaged group. Targeted enhanced training is warranted for personnel in community hospitals, those with low seniority (<5 years), and nursing professionals, with a particular need to improve knowledge translation efficiency (reflected by a lower increase in practice OR compared to knowledge). For detailed results, refer to Table 5.

**Table 5:** Practice scores and influencing factors.

Demographic characteristic	Practice scores	*P*-value	Variable	Active practice OR (95% CI)	*P*-value
Age (yr)			Age (Ref: <25 yr)		
<25 yr	35.25 ± 6.32	<.001			
25–35 yr	37.45 ± 5.88		25–35 yr	1.37 (0.91–2.06)	.132
36–45 yr	38.63 ± 5.61		36–45 yr	2.03 (1.30–3.17)	.002
>45 yr	39.79 ± 4.57		>45 yr	2.87 (1.74–4.74)	<.001
Gender			Gender (male vs female)	0.79 (0.56–1.11)	.172
Male	38.12 ± 5.87	.424			
Female	37.68 ± 6.01				
Professional title			Title (Ref: Assistant)		
Assistant	34.50 ± 6.31	<.001			
Junior	36.92 ± 6.15		Junior	3.88 (2.05–7.35)	<.001
Intermediate	38.95 ± 5.12		Intermediate	2.78 (1.51–5.13)	.001
Senior	41.88 ± 4.23		Senior	2.05 (1.12–3.76)	.020
Professional category			Prof Cat. (Ref: Nursing)		
Clinical medicine	38.26 ± 5.43	<.001	Clinical medicine	1.22 (0.82–1.82)	.328
Traditional Chinese Med	39.82 ± 4.87		Traditional Chinese Med	1.89 (1.17–3.06)	.009
Integrated TCM-WM	41.51 ± 4.12		Integrated TCM-WM	3.17 (1.98–5.08)	<.001
Nursing	40.13 ± 5.57				
Hospital level			Hospital level (Ref: Community)		
Community	35.12 ± 6.47	<.001			
Secondary	37.95 ± 5.43		Secondary	1.75 (1.15–2.66)	.009
Tertiary	39.79 ± 4.57		Tertiary	2.38 (1.58–3.58)	<.001
Years of practice (yr)			Years Pract. (Ref: <5 yr)		
<5 yr	36.05 ± 6.48	<.001			
5–10 yr	37.53 ± 5.79		5–10 yr	1.47 (0.95–2.27)	.083
11–15 yr	38.81 ± 5.54		11–15 yr	2.06 (1.29–3.29)	.002
>15 yr	40.06 ± 5.22		>15 yr	2.89 (1.72–4.86)	<.001

CI = confidence interval, OR = odds ratio, TCM-WM = Traditional Chinese and Western medicine.

### 3.7. Correlation analysis of knowledge, attitude, and practice

Pearson correlation analysis revealed significant positive correlations between all pairs of KAP dimensions (all *P* < .001). The Knowledge-Practice correlation was the strongest (*R* = 0.612), indicating that knowledge enhancement is a core lever for improving clinical practice. The Attitude-Practice correlation was moderately strong (*R* = 0.593). The efficiency of translating knowledge into practice (*R* = 0.612) was higher than translating knowledge into attitude (*R* = 0.484), suggesting a need for attitude interventions to strengthen knowledge application (Table 6).

**Table 6:** Correlation analysis of knowledge, attitude, and practice.

	Knowledge	Attitude	Practice
Knowledge	1.00	–	–
Attitude	0.484	1.00	–
Practice	0.612	0.593	1.00

Note: *r* = Pearson correlation coefficient, range [−1,1], absolute value indicates strength.

### 3.8. Multiple logistic regression analysis of physicians’ KAP scores

Multiple logistic regression analysis for physicians, adjusting for gender, age, hospital level, etc, examined the impact of demographics on high knowledge, positive attitude, and active practice groups. Gender showed no significance for Knowledge (OR = 0.92, *P* = .781) or Attitude (OR = 1.15, *P* = .549). Secondary hospitals showed borderline significance for Knowledge (*P* = .084) and Practice (*P* = .072). Integrated TCM-WM showed no significant association with Knowledge (OR = 0.78, *P* = .432) or Attitude (OR = 0.85, *P* = .584). TCM physicians significantly outperformed Clinical Medicine physicians in all KAP dimensions (OR > 3.75). Integrated TCM-WM physicians showed an advantage only in Practice (OR = 1.97). Tertiary hospitals, high titles, and senior physicians formed the KAP advantage group, with title gradient effects being most pronounced (Senior title Practice OR = 6.01). Male physicians showed significantly higher practice initiative than females (OR = 1.88), but no gender difference existed for knowledge or attitude, suggesting practice behavior is moderated by non-cognitive factors (Table 7).

**Table 7:** Multiple logistic regression analysis of physicians’ KAP scores.

Demographic characteristic	High knowledge OR (95% CI)	*P*-value	Positive attitude OR (95% CI)	*P*-value	Active practice OR (95% CI)	*P*-value
Gender (male vs female)	0.92 (0.51–1.66)	.781	1.15 (0.73–1.81)	.549	1.88 (1.12–3.16)	.017
Age (per 5-yr increase)	2.84 (1.32–6.11)	.008	1.27 (0.68–2.38)	.452	3.01 (1.45–6.25)	.003
Hospital level (Ref: Community)						
Tertiary	4.62 (2.15–9.93)	<.001	1.97 (1.04–3.73)	.038	3.25 (1.69–6.25)	<.001
Secondary	1.85 (0.92–3.71)	.084	1.42 (0.78–2.58)	.249	1.78 (0.95–3.33)	.072
Prof Cat. (Ref: Clin. Med.)						
Traditional Chinese Med. (TCM)	3.75 (1.82–7.72)	<.001	4.10 (2.11–7.96)	<.001	5.22 (2.58–10.56)	<.001
Integrated TCM-WM	0.78 (0.42–1.45)	.432	0.85 (0.48–1.52)	.584	1.97 (1.12–3.47)	.019
Title (senior vs junior)	5.33 (2.58–11.02)	<.001	3.28 (1.72–6.25)	<.001	6.01 (2.89–12.50)	<.001
Years Pract. (>10 yr vs <5 yr)	3.12 (1.55–6.28)	.001	2.25 (1.15–4.41)	.018	3.45 (1.72–6.92)	<.001

CI = confidence interval, OR = odds ratio, TCM-WM = traditional Chinese and Western medicine.

### 3.9. Multiple logistic regression analysis of other medical staff KAP scores

Analysis for non-physician staff (nurses/technicians), adjusting for confounders, showed: Secondary hospitals approached significance for Practice (OR = 1.85, *P* = .062), 5 to 10 years experience was borderline significant for Knowledge (OR = 1.72, *P* = .084). Age and gender showed no significance for Knowledge/Attitude (*P* > .05). Tertiary hospital staff had significant advantages in Knowledge (OR = 2.95) and Practice (OR = 2.78), but Attitude was unaffected by hospital level (*P* = .249). Females had significantly higher practice initiative than males (OR = 2.05), reflecting the operational nature of nursing roles. Staff with >10 years experience significantly outperformed juniors (<5 years) in all KAP dimensions (OR > 1.98), while the 5 to 10 years group showed no significant improvement, suggesting a threshold effect around 10 years. Senior titles significantly enhanced Knowledge and Practice, but Attitude was more associated with Intermediate titles (OR = 2.10), indicating role differentiation (Table 8).

**Table 8:** Multiple logistic regression analysis of other medical staff KAP scores.

Demographic characteristic	High knowledge OR (95% CI)	*P*-value	Positive attitude OR (95% CI)	*P*-value	Active practice OR (95% CI)	*P*-value
Gender (male vs female)	1.25 (0.73–2.14)	.412	0.88 (0.52–1.49)	.638	2.05 (1.21–3.47)	.008
Age (per 5-yr increase)	1.56 (0.82–2.97)	.176	0.97 (0.55–1.81)	.926	1.72 (0.92–3.22)	.089
Hospital level (Ref: Community)						
Tertiary	2.95 (1.52–5.73)	.001	1.42 (0.78–2.58)	.249	2.78 (1.49–5.19)	.001
Secondary	1.62 (0.85–3.09)	.142	1.18 (0.65–2.14)	.584	1.85 (0.97–3.52)	.062
Title (Ref: Junior)						
Senior	1.88 (1.02–3.47)	.043	1.52 (0.85–2.72)	.306	2.25 (1.25–4.05)	.007
Intermediate	1.42 (0.76–2.65)	.271	2.10 (1.15–3.83)	.028	1.72 (0.93–3.18)	.084
Years Pract. (Ref: <5 yr)						
5–10 yr	1.72 (0.93–3.18)	.084	1.37 (0.75–2.50)	.306	2.01 (1.10–3.68)	.024
>10 yr	2.15 (1.15–4.02)	.017	1.98 (1.08–3.63)	.028	2.45 (1.32–4.55)	.004

CI = confidence interval, OR = odds ratio.

## 4. Discussion

This cross-sectional survey evaluated the current Knowledge, Attitudes, and Practices (KAP) of healthcare professionals across multi-tiered medical institutions in Zhengzhou regarding the integrated traditional Chinese and Western medicine treatment for AIS. We achieved a valid response rate of 88.23%, and the absence of significant differences in key characteristics between respondents and non-respondents indicates good representativeness of the sample for the target population. The participants were predominantly young, female, with junior professional titles, and covered a spectrum from tertiary hospitals to community health centers. This profile likely reflects the general composition of frontline personnel in the Chinese healthcare system, but it also suggests that the study findings are particularly influenced by the perceptions and behaviors of this core demographic. Our research revealed 3 key findings: Disparities in healthcare resources across institution levels led to an “inverted pyramid” distribution in KAP performance; Professional background is a critical determinant of cognitive integration and potential for practice; and The translation of knowledge into practice varies significantly among groups, with attitude playing a pivotal moderating role. These findings will be discussed in depth in the following section.

### 4.1. Imbalanced healthcare resources and the “inverted pyramid” dilemma in KAP

The primary finding of this study is that hospital level is one of the strongest predictors of KAP levels. Healthcare professionals in tertiary hospitals had significantly greater odds of being in the high-knowledge group (OR = 2.74) and the proactive-practice group (OR = 2.38) compared to those in community hospitals. This suggests that disparities in hospital level, manifested through resource allocation, technological accumulation, and systematic management, form a core driver of KAP stratification. This may be attributed to the more comprehensive stroke center infrastructure and higher accessibility of Chinese medicine injections in tertiary hospitals within Zhengzhou, facilitating standardized treatment.^[14,15]^ This disparity likely stems from uneven resource distribution: tertiary hospitals typically possess a more complete range of Chinese medicine injections, acupuncture equipment, and specialized stroke units, providing the material foundation for integrated practice. In contrast, community hospitals, despite undertaking nearly 40% of initial AIS diagnoses, commonly face challenges such as low thrombolysis rates, lack of thrombectomy qualifications (coverage rate in Zhengzhou community hospitals is only 12.1%), and insufficient authorization for Traditional Chinese Medicine technical procedures. Consequently, although community healthcare workers demonstrate a relatively high awareness of basic theories (e.g. the thrombolytic time window), the lack of practical conditions directly contributes to their lower KAP scores, particularly in the practice dimension. The innovativeness of this study lies not only in quantifying this hierarchical disparity but also in confirming the independent influence of hospital level through a multiple regression model adjusted for other variables. This indicates that enhancing the effectiveness of the regional stroke prevention and treatment network must focus on resolving the structural contradictions in resource allocation.

### 4.2. Professional differentiation: the knowledge integration advantage of TCM-WM and the practice potential of nursing staff

Our second key finding is the significant differentiation based on professional background. Professionals in Integrated Traditional Chinese and Western Medicine led comprehensively across all KAP dimensions (Knowledge: 28.64 ± 2.15; Attitude: 52.69 ± 3.18; Practice: 41.51 ± 4.12), with 3.25 times the odds of high knowledge compared to nursing staff. This advantage is rooted in their unique training model. In China, Integrated TCM-WM professionals receive systematic education in both Western medicine (including anatomy, physiology, pathophysiology) and Traditional Chinese Medicine (including TCM theory, Chinese materia medica, acupuncture). This integrated training enables them to synthesize knowledge and develop a “disease-syndrome combination” diagnostic and therapeutic mindset, thereby demonstrating unique advantages in managing diseases like AIS, which requires acute-phase Western intervention and recovery-phase comprehensive management.^[16,17]^ This study provides empirical support for the clinical value of this training model through detailed KAP data. Research by Zhai Y et al^[18]^ on cases like using Shuanglu Tongnao formula for convalescent stroke also corroborates that integrated TCM-WM can achieve synergistic “1 + 1 > 2” effects through multi-target regulation (e.g. anticoagulation, anti-inflammation, neuroprotection). Similarly, Gou LK et al^[19]^ reported that applying acupuncture combined with Chinese herbal decoctions in patients with acute severe stroke not only significantly improved intestinal mucosal barrier function, immune function indicators, and brain nerve-related scores but also yielded good overall outcomes, promoting recovery.

In interesting contrast is the case of nursing staff. They had the lowest knowledge scores (23.13 ± 3.67) but demonstrated prominent practice scores (40.13 ± 5.57). This phenomenon needs to be understood within the context of nursing education in China, where the curriculum focuses more on Western nursing procedures and monitoring skills, with relatively limited instruction on TCM theory and herbal mechanisms, potentially leading to their knowledge gap.^[20]^ However, nurses are the primary executors of medical orders and the group with the most frequent patient contact. Their proficient operational skills (“procedural memory”) and frequent clinical exposure equip them with rich practical experience. Furthermore, subgroup analysis revealed that among non-physician staff, females had significantly higher odds of proactive practice than males (OR = 2.05). This might stem from the female-dominated composition of the nursing workforce and potential female advantages in soft skills like communication and patience, which are crucial for patient education and daily care, leading to more active practice. This finding innovatively highlights the underutilized practical potential of the nursing team in integrated stroke care, suggesting they could serve as vital bridges for knowledge translation.

### 4.3. The knowledge-to-practice translation mechanism: the moderating role of attitude

Correlation analysis indicated a strong positive correlation between knowledge and practice (*R* = 0.612), which was higher than the knowledge-attitude correlation (*R* = 0.484). However, regression models revealed a more complex picture. Among physicians, attitude did not significantly directly predict practice; their clinical behavior is likely driven more by technical-rational factors like evidence-based guidelines and professional promotion (e.g., senior physicians OR = 6.01 for practice). Conversely, among other healthcare staff like nurses, attitude played a more substantial moderating role (e.g., intermediate-level nurses OR = 2.10 for attitude), meaning a positive attitude might enhance their motivation to translate existing knowledge into practice. We cautiously use the term “moderating” rather than “mediating” here, as formal mediation effect testing was not conducted. This finding aligns with the Theory of planned behavior and refines the understanding of behavioral drivers across different healthcare roles: physicians’ prescribing decisions rely more on objective evidence, while nurses’ operational execution is more influenced by subjective beliefs and willingness.^[21,22]^ Therefore, mere knowledge dissemination may have limited impact in community hospitals; it needs to be complemented by attitude intervention strategies. For physicians, this could involve simulated training for thrombolysis–thrombectomy bridging therapy to strengthen technical confidence. For nurses, sharing patient rehabilitation case studies and demonstrating imaging evidence of acupuncture improving hemiplegia could be effective.

### 4.4. Strengths and limitations

The primary strength of this study lies in its multi-stage, stratified sampling strategy, which ensured good coverage of multi-tiered medical institutions from tertiary hospitals to community centers. The high valid response rate of 88.23%, coupled with the non-response bias analysis, collectively supports the sample’s representativeness of the target population. Secondly, this study is the first to focus specifically on healthcare professionals’ KAP regarding integrated TCM-WM for AIS and conducted physician/non-physician subgroup analyses, providing nuanced insights into behavioral differences among roles. However, as a cross-sectional survey, the inherent design limits causal inference. Furthermore, reliance on self-reported questionnaire data is susceptible to social desirability bias, potentially leading to overestimation of results, especially for practice behaviors. Finally, although Zhengzhou, as a National Central City, is representative, the single-region nature of the study somewhat limits the direct generalizability of the conclusions to other regions with different healthcare policies or cultural contexts. These limitations suggest the need for future multi-center longitudinal studies incorporating objective behavioral indicators to further validate and deepen the current findings.

## 5. Conclusions

In summary, this study systematically delineates the current KAP landscape of healthcare professionals in Zhengzhou regarding integrated TCM-WM treatment for AIS and identifies 3 core dimensions influencing KAP levels: resource hierarchy, professional differentiation, and attitude moderation. The conclusions indicate that future optimization of the regional stroke prevention and treatment network requires an integrated tripartite strategy: in resource allocation, establishing “tertiary-community” stroke green channels and delegating simplified TCM techniques; in talent development, strengthening the knowledge base of community and nursing staff and authorizing senior nurses to perform standardized TCM procedures; in mechanism building, designing role-specific training programs, reinforcing technical confidence for physicians, and using rehabilitation cases to stimulate attitude shifts among nurses. Future research should validate the effectiveness of these strategies through intervention experiments and delve deeper into the specific cognitive and neural mechanisms underlying the translation of knowledge into practice.Supplemental digital content “Appendix” is available for this article (https://links.lww.com/MD/Q730).

## Author contributions

**Conceptualization:** Yiwei Zhang.

**Data curation:** Haobo Gao.

**Formal analysis:** Hongtu Tan, Jiabin Wang.

**Funding acquisition:** Tao Wu.

**Investigation:** Jiabin Wang.

**Methodology:** Guofang Yang.

**Project administration:** Dongyi Yang, Tao Wu.

**Resources:** Dongyi Yang.

**Supervision:** Yaheng Zhao, Lei Xie, Tao Wu.

**Validation:** Yaheng Zhao, Lei Xie, Tao Wu.

**Writing – original draft:** Yiwei Zhang, Haobo Gao.

**Writing – review & editing:** Hongtu Tan, Tao Wu.

## Supplementary Material


