# Supplementary material for: KAP survey on integrated traditional Chinese and western medicine for ischemic stroke among healthcare workers in Zhengzhou City: A cross-sectional study based on multi-tiered healthcare institutions
Source: Medicine (Baltimore). 2025 Nov 28;104(48):e46069. doi: 10.1097/MD.0000000000046069 (PMC12662467; doi:10.1097/MD.0000000000046069)
Supplement: Supplementary file 1 [file medi-104-e46069-s001.docx]

***List of Abbreviations**

| **Abbreviation** | **Abbreviation** |
| --- | --- |
| AIS | Acute Ischemic Stroke |
| KAP | Knowledge, Attitude, Practice |
| rt-PA | recombinant tissue plasminogen activator |
| S-CVI | Scale-level Content Validity Index |
| I-CVI | Item-level Content Validity Index |
| EFA | Exploratory Factor Analysis |
| KMO | Kaiser-Meyer-Olkin |
| ICC | Intraclass Correlation Coefficient |
| OR | Odds Ratio |
| CI | Confidence Interval |
